# Natural Antioxidants and Aquatic Animal Health—2nd Edition

**DOI:** 10.3390/antiox15091139

**Published:** 2026-09-09

**Authors:** Rui Jia

**Affiliations:** Key Laboratory of Integrated Rice-Fish Farming Ecology, Ministry of Agriculture and Rural Affairs, Freshwater Fisheries Research Center, Chinese Academy of Fishery Sciences, Wuxi 214081, China; jiar@ffrc.cn

## 1. Introduction

Sustainable aquaculture demands not only high production efficiency but also the maintenance of physiological health in farmed aquatic animals [1,2]. Yet as stocking density rises, water quality often degrades and crowding stress becomes more frequent, which may lead to a higher incidence of disease [3,4]. Under such pressure, the antioxidant defense system acts as a frontline barrier [5,6]. But it is not unbreakable. When stress persists or intensifies, the balance between reactive oxygen species (ROS) generation and scavenging would be disturbed, which causes suppression of the antioxidant defense system [7]. The imbalance of redox homeostasis further leads to other physiological abnormalities, increasing the susceptibility of aquatic animals to environmental challenges and pathogens [8]. It is therefore critical to evaluate how environmental challenges induce oxidative stress, as well as to develop practical strategies to improve the antioxidant capacity of farmed aquatic animals.

A growing body of evidence points to bioactive compounds as promising feed additives that enhance antioxidant capacity and support aquatic animal health [9]. These compounds have also been reported to modulate immune responses, inflammatory signaling, nutrient metabolism, and intestinal function [10,11]. These reports indicate that dietary antioxidants may exert beneficial effects not only through direct antioxidant activity but also by modulating several physiological processes. However, for aquatic animals, the specific physiological roles and underlying mechanisms remain to be further explored.

The Special Issue “Natural Antioxidants and Aquatic Animal Health—2nd Edition” aims to provide new insights into the role of antioxidants in maintaining aquatic animal health. It highlights key molecular mechanisms of some feed additives with antioxidant properties and their potential applications in aquaculture. It also focuses on the critical role of antioxidant defense systems in the responses of aquatic animals to environmental stressors. Here, we provide an overview of the 11 original research articles in this Special Issue.

## 2. Overview of Published Articles

Dietary supplementation with antioxidants can enhance antioxidant defense and help maintain physiological homeostasis and health in aquatic animals. In this Special Issue, several studies investigated the antioxidative effects of certain feed additives and further explored the underlying molecular mechanisms. Dietary vitamin B6 enhanced antioxidant capacity in juvenile largemouth bass (*Micropterus salmoides*), as indicated by increased antioxidant enzyme activities and upregulated antioxidant-related gene expression. Its beneficial effects on intestinal antioxidant defense were also associated with regulation of the Keap1/Nrf2-ARE signaling pathway [Contribution 1]. In juvenile blunt snout bream (*Megalobrama amblycephala*) fed an all-plant-protein diet, dietary methionine supplementation improved growth performance and hepatic antioxidant defense and maintained mitochondrial function. It also altered hepatic m6A methylation and the expression of autophagy- and apoptosis-related genes to regulate metabolic and cellular homeostasis [Contribution 2]. In red swamp crayfish (*Procambarus clarkii*), Guo et al. [Contribution 3] evaluated the ameliorative effect of phosphorus supplementation. They found that increasing dietary phosphorus levels to 1.40-1.52% effectively alleviated growth retardation, oxidative damage, and lipid metabolic disturbances caused by high-level bacterial protein. Similarly, appropriate supplementation with chenodeoxycholic acid and ursodeoxycholic acid was applied to little yellow croaker (*Larimichthys polyactis*) under nutritional imbalance. The intervention improved growth performance, modulated thyroid hormone metabolism, and strengthened intestinal antioxidant capacity [Contribution 4].

Besides nutritional intervention, several studies examined the molecular and metabolic changes associated with oxidative stress in fish and crab. Yang et al. [Contribution 5] examined the effects of oxidized fish oil on blunt snout bream muscle. They found that it induced oxidative stress, as evidenced by increased malondialdehyde (MDA) levels and reduced superoxide dismutase (SOD) and glutathione peroxidase (GPx) activities. The resulting muscle deterioration was closely associated with miR-144-mediated Nrf2 signaling. Similarly, in the muscle of common carp (*Cyprinus carpio*), H_2_O_2_-induced oxidative stress caused marked metabolic and transcriptional changes [Contribution 6]. These changes mainly involved oxidative phosphorylation, adipocytokine signaling, and PPAR signaling. Feng et al. [Contribution 7] further reported that H_2_O_2_-induced oxidative stress elicited gill damage in the Chinese mitten crab (*Eriocheir sinensis*) and concurrently provoked a compensatory antioxidant response. It also altered ion transport and osmoregulation and upregulated the expression of genes involved in apoptosis and autophagy. A comparable pattern was observed in GIFT tilapia (*Oreochromis niloticus*), where microcystin-LR injection induced oxidative stress and liver damage. These effects were accompanied by intestinal microbiota dysbiosis and short-chain fatty acid metabolism disturbances [Contribution 8]. These studies illustrate that oxidative stress can simultaneously affect antioxidant defense, metabolic pathways and tissue-specific physiological functions.

The antioxidant defense system contributes substantially to the adaptation of aquatic animals to environmental fluctuations. In large yellow croaker (*Larimichthys crocea*) exposed to varying pH levels, pH 7.8 was associated with the lowest lipid peroxidation and highest antioxidant enzyme activities, suggesting this pH level as more favorable for the species [Contribution 9]. In response to heat stress, Hong Kong oyster (*Magallana hongkongensis*) triggered antioxidant, apoptotic, inflammatory, and heat shock protein-related pathways. However, these protective responses were disrupted when temperatures exceeded 35 °C, leading to survival impairment [Contribution 10]. In turbot (*Scophthalmus maximus*), Liu et al. [Contribution 11] reported a similar pattern. High-temperature stress induced the loss of daily rhythmic regulation of SOD and accumulation of MDA. They also found that heat stress poses a destructive threat to circadian integrity. These studies suggest that the antioxidant defense system offers protection under moderate stress; however, when stress becomes excessive, the system may be overwhelmed, ultimately resulting in tissue damage.

## 3. Conclusions

Taken together, this collection highlights the potential application value of dietary antioxidants in supporting aquatic animal health. It also offers new insights into the mechanism by which the antioxidant defense system responds to environmental stressors. However, given the structural diversity of natural antioxidants and the physiological differences across aquatic species, the current knowledge remains incomplete. Furthermore, the safety of these compounds for different farmed aquatic animals will require more systematic evaluation. We thank all contributors for their valuable studies, and hope this Special Issue will stimulate continued research on the relationship between natural antioxidants and aquatic animal health.
